# A Factor Analysis of the COMFORTneo Scale: What Are We Measuring?

**DOI:** 10.1002/pne2.70033

**Published:** 2026-05-14

**Authors:** Erik Koning, Arend F. Bos, Elisabeth M. W. Kooi, Alexander J. de Tempe, Nienke H. van Dokkum

**Affiliations:** ^1^ Division of Neonatology, Department of Pediatrics Beatrix Children's Hospital, University Medical Center Groningen Groningen the Netherlands

**Keywords:** COMFORTneo scale, preterm infants, unidimensionality

## Abstract

Extremely preterm infants (EPT; < 26 weeks) are often exposed to painful and stressful procedures, and in clinical practice it is difficult to distinguish pain from stress. This study investigated whether the COMFORTneo scale can differentiate between these constructs in EPT and older preterm infants (PT; 26–36 weeks' gestational age). We analyzed COMFORTneo scores from 1021 preterm infants admitted to a level IV NICU between 2018 and 2022. Infants were divided into four subgroups: EPT ventilated, EPT non‐ventilated, PT ventilated, and PT non‐ventilated. Exploratory and confirmatory factor analyses were used to assess the underlying structure of the scale. In addition, a panel of 11 NICU professionals completed a short questionnaire regarding the constructs represented by COMFORTneo items. Exploratory factor analysis indicated a unidimensional structure for EPT infants, whereas two factors emerged in PT infants, with interfactor correlations of *r* = 0.78 in ventilated infants and *r* = 0.63 in non‐ventilated infants. Confirmatory factor analysis supported these structures, with comparative fit index (CFI) values ≥ 0.96 and root mean square error of approximation (RMSEA) ranging from 0.070 to 0.091. Experts' interpretations of the constructs associated with the identified factors varied across respondents. Overall, the COMFORTneo scale appears to capture a single construct in EPT infants and two related constructs in PT infants. However, NICU professionals did not consistently distinguish between these constructs in practice. These findings highlight the importance of developmentally sensitive interpretation and caution against relying solely on total COMFORTneo scores.

## 1 Introduction

Preterm infants, especially those born extremely preterm (EPT < 26 weeks of gestation), are often repeatedly subjected to painful, stressful, or both interventions in the Neonatal Intensive Care Unit (NICU) [1, 2, 3]. The earlier an infant is born, the more likely they are to undergo interventions compared with older preterm infants (26–36 weeks of gestation) [4, 5, 6]. Although repeated interventions in the NICU are usually inevitable, they can have adverse effects on neurological and behavioral development [5, 6, 7].

The potential adverse effects on neurodevelopment underscore the importance of adequate pain and stress management [5]. Nevertheless, pain and stress management are challenging due to the inability of infants to self‐report [5, 8, 9]. Furthermore, it is essential to note that preterm infants are a heterogeneous group. Neurological immaturity, in particular, will be more prevalent in EPT infants than in PT infants [4]. This neurological immaturity in EPT infants may lead to less differentiated behavioral responses, which affect the way pain and distress are expressed and measured [9]. Reliable observational scales that account for developmental differences between EPT and PT infants are needed [5].

Effective pain management should consider differences in neurodevelopment and also requires an understanding of how pain and stress manifest in different subgroups of preterm infants [10]. Although pain and stress are often associated with similar behaviors, they arise from different underlying mechanisms [11]. Nevertheless, different mechanisms underlie this. Pain results from tissue damage, such as skin lesions, while stress results from a disruption of homeostasis [8, 9, 12]. Adequately differentiating between pain and stress can contribute to effective treatment [5, 6, 7, 8].

Most neonatal assessment tools focus primarily on pain and offer only limited insight into stress [9, 13]. It remains unclear whether these tools are suitable for EPT infants [14]. A few tools attempt to separate the constructs of pain and stress, but their validity and reliability remain uncertain [10, 13]. The COMFORTneo scale was developed to measure both pain and stress using seven behavioral indicators chosen for clinical relevance [15, 16]. It correlates moderately to strongly with numeric rating scales (*r* = 0.52 for pain; *r* = 0.70 for stress) and shows good internal consistency (Cronbach's α 0.84–0.88) [15]. However, Cronbach's α and correlation coefficients do not clarify which latent construct the scale measures [17, 18].

This study aims to identify the latent structure of the COMFORTneo scale, examine item relationships to these constructs, and determine whether factor structures differ between EPT and PT infants.

## 2 Methods

We collected COMFORTneo scale scores from all preterm infants born between 24 and 36 weeks of gestation admitted to our level IV NICU from January 1, 2018, to December 31, 2022. Infants were divided into four subgroups: EPT ventilated, EPT non‐ventilated, PT ventilated, and PT non‐ventilated. This subdivision was necessary because the COMFORTneo scale differentiates between ventilated and non‐ventilated infants. It assesses respiratory response in ventilated infants and crying in non‐ventilated infants [15].

### 2.1 COMFORTneo Scale

The Comfortneo scale consists of seven items, namely: alertness, agitation, muscle tension, facial tension, body movements, and respiratory response (for ventilated infants) or crying (for non‐ventilated infants) [15]. Each item was rated on a five‐point scale, resulting in a total score ranging from 6 (no discomfort) to 30 (maximum discomfort). Each preterm infant was scored during a two‐minute observation by a NICU nurse. According to local protocol, one score was recorded per nursing shift (which lasted 8 h per day, resulting in three scores per day). All COMFORTneo scores were recorded by NICU nurses trained in the use of the scale, ensuring consistency and reliability of assessments. In addition to routine assessments, event‐triggered COMFORTneo observations were performed when clinically indicated, for example following potentially painful or stressful procedures or changes in clinical condition. All assessments were ideally conducted during calm periods with minimal environmental noise and at least 3 min after potentially painful or stressful procedures. For this study, scores over the first 5 days were calculated as a weighted average score per infant to summarize repeated COMFORTneo assessments into a single value per infant, as the number of assessments differed between infants. Missing data were defined as infants who did not have any COMFORTneo scale scores during the first five postnatal days. Infants with at least one score in this period were included in the calculation of the weighted average.

### 2.2 Expert Questionnaire

To supplement the statistical analysis, we administered a short questionnaire to NICU health professionals with experience in neonatal pain and stress assessment, including a neonatologist, two nurse practitioners, six NICU nurses, and two music therapists. Participants were asked to indicate which underlying constructs they believed belonged to the factors identified through factor analysis, based on the corresponding COMFORTneo items. The questionnaire was completed individually, and the responses were summarized and analyzed for recurring patterns and similarities.

### 2.3 Analysis

The baseline characteristics (gestational age, birth weight, sex, and respiratory support) were extracted from electronic patient records and described using descriptive statistics.

To assess whether the COMFORTneo scale measures a unidimensional or multidimensional construct across infant subgroups, we performed exploratory factor analysis (EFA) separately for each group. EFA was chosen because the COMFORTneo scale was adapted from the original COMFORT scale, and prior research suggests that it encompasses multiple underlying constructs, including pain and stress [15]. EFA identifies the number of factors and the items loading onto each factor. Normality of the data was assessed with the Shapiro–Wilk test [19, 20]. A correlation matrix was created to evaluate relationships between items [21, 22]. We applied the Promax rotation, an oblique method that allows factors to correlate, consistent with the theoretical overlap between pain‐ and stress‐related behaviors [15, 22, 23]. Based on the rotated factor loadings, a conceptual model describing factors and their relationships was constructed [23, 24]. Model fit was evaluated using the Root Mean Square Residual (RMSR) and the Root Mean Square Error of Approximation (RMSEA), with RMSR ≤ 0.05 and RMSEA ≤ 0.08 considered acceptable [24]. Ultra Heywood cases, such as negative variance estimates, indicate potential issues with model specification and were checked to ensure the validity of the factor analysis [25].

Confirmatory factor analysis (CFA) was carried out after the EFA to validate the best factor structures found within each subgroup [22, 26]. Items that cross‐loaded on more than one factor were removed to preserve a clear and intelligible factor structure. Items were then assigned to factors based on loadings of 0.40 or higher to ensure significant associations. In CFA, model fit was assessed using the Tucker‐Lewis Index (TLI), Comparative Fit Index (CFI), and RMSEA. When the RMSEA was less than 0.08 and the TLI and CFI were both greater than 0.95, we deemed the model fit to be satisfactory [24]. Cronbach's α and Composite Reliability (CR) were used to assess internal consistency; values of 0.70 or higher were considered adequate. EFA and CFA were both performed on the same dataset, given the available sample size.

Sampling adequacy was evaluated using the Kaiser‐Meyer‐Olkin measure, with values greater than 0.60 considered acceptable and Bartlett's test of sphericity (*p* < 0.05). All analyses were conducted in R (version 4.3.1) using the psych and lavaan packages.

## 3 Results

### 3.1 Sample Characteristics

We collected data from 1072 preterm infants who were admitted between 2018 and 2022. After 51 cases were discarded due to missing data, 106 of the 1021 infnats in the final sample were identified as EPT infants (Table 1). The COMFORTneo scores were averaged over the first 5 days of life. For each subgroup, sampling adequacy was confirmed with Bartlett's test of sphericity (*p* < 0.001) and the Kaiser‐Meyer‐Olkin measure (all > 0.60), indicating suitability for factor analysis.

**TABLE 1:** Baseline characteristics of the entire group of preterm infants.

		Total	PT infants	EPT infants
Gestational age (weeks)		24–36 *N* = 1021	26 + 1–36 *n* = 915 (90%)	24–26 + 0 *n* = 106 (10%)
Sex (male)		*N* = 477 (47%)	*n* = 431 (47%)	*n* = 46 (43%)
Birth weight (gram)		995	1280	760
Median (IQR)		(790–1448)	(950–1655)	(680–830)
Type of respiration response during COMFORTneo scale scoring	Ventilated	*N* = 233 (23%)	*N* = 174 (19%)	*N* = 59 (56%)
	Non (invasively)‐ventilated	*N* = 788 (77%)	*N* = 741 (81%)	*N* = 49 (44%)
Distribution of COMFORTneo scale scores. Median (IQR)	Ventilated		12 (10–14)	10 (9–13)
	Non (invasively)‐ventilated		13 (10–14)	11 (10–13)

Abbreviations: EPT, extremely preterm infants; IQR, interquartile range; PT, preterm infants.

### 3.2 Exploratory Factor Analysis (EFA)

The results of the EFA and CFA for all EPT and PT subgroups are presented in Table 2. We conducted EFA separately within the four predefined subgroups: EPT ventilated, EPT non‐ventilated, PT ventilated, and PT non‐ventilated. In both EPT subgroups we found an unidimensional structure: all relevant items loaded onto one factor, suggesting that the COMFORTneo scale reflects one overarching construct. We did not apply rotation because we found only one factor. Conversely, a two‐factor solution was found in the PT ventilated and PT non‐ventilated subgroups. We used Promax rotation, an oblique technique that allows for factor correlation. The items loaded clearly on two factors with low cross‐loadings. After rotation, a moderate to strong interfactorial correlation was found (*r* = 0.78 for ventilated patients, *r* = 0.63 for non‐ventilated patients). All subgroups' model fit indices were deemed generally acceptable; the PT ventilated subgroup had a slightly higher RMSEA value, ranging from 0.070 to 0.091, and the RMSR was below the 0.08 threshold. Although RMSEA for one factor in the PT ventilated subgroup exceeded conventional thresholds (RMSEA = 0.20), overall model fit indices were acceptable, and the two‐factor structure remained theoretically and empirically supported.

**TABLE 2:** Results of EFA and CFA for all subgroups of EPT and PT infants.

EFA per subgroup						
Aspect	EPT Ventilated (*N* = 59)	EPT Non‐Ventilated (*N* = 47)	PT Ventilated (*N* = 174)		PT Non‐Ventilated (*N* = 741)	
Factors	F1	F1	F1	F2	F1	F2
Items per factor and their loadings	1 alertness (0.89) 2 agitation (0.95) 3 respiratory response (0.89) 5 facial tension (0.79) 6 muscle tone (0.50) 7 movement (0.61)	1 alertness (0.72) 2 agitation (0.95) 4 crying (0.32) 5 facial tension (0.60) 6 muscle tone (0.74) 7 movement (0.75)	1 alertness (0.54) 2 agitation (0.76) 6 muscle tone (0.66) 7 movement (0.88)	3 respiratory response (0.94) 5 facial tension (0.46)	1 alertness (0.62) 2 agitation (0.73) 4 crying (0.91) 6 muscle tone (0.76)	5 facial tension (0.56) 7movement (0.90)
Rotation method	None	None	Promax		Promax	
Interfactor Correlation	—	—	0.78		0.63	
RMSR	< 0.05	< 0.05	F1: 0.0870α F2: 0.0006		F1: < 0.06 F2: < 0.06	
RMSEA	< 0.06	< 0.06	F1: 0.20 F2: < 0.06		F1: < 0.06 F2: < 0.06	
CFA per subgroup						
*χ* ^2^(df), p	85.34 (40), *p* < 0.001	103.44 (40), *p* < 0.001	325.01 (30), *p* < 0.001		265.23 (30), *p* < 0.001	
RMSEA (90% CI)	0.088 (0.052–0.13)	0.091 (0.05–0.13)	0.070, (0.060–0.080)		0.075, (0.065–0.085)	
CFI/TLI	0.97/0.96	0.97/0.96	0.98/0.97		0.98/0.97	
Cronbach's α	0.85–0.92	0.84–0.91	0.83–0.90		0.85–0.91	
Composite Reliability	0.86–0.93	0.85–0.92	0.84–0.91		0.86–0.92	
Interfactor Correlation (CFA)	—	—	0.61		0.65	

Abbreviations: CFA, confirmatory factor analysis; CFI, comparative fit index; df, degrees of freedom; EFA, exploratory factor analysis; F, factor; *N*, sample size; *p*, significance level; RMSEA, root mean square error of approximation; RMSR, root mean square residual; TLI, tucker‐lewis index; χ^2^, chi‐square.

### 3.3 Confirmatory Factor Analysis (CFA)

We performed a CFA to validate these factor structures. We used one‐factor models for the EPT subgroups and two‐factor models for the PT subgroups. Items with cross‐loadings were excluded, and the others were assigned to factors based on their most substantial loadings (≥ 0.40). The model fit was good across all CFA models, with RMSEA values ranging from 0.070 to 0.091 and CFI/TLI consistently above 0.96. Cronbach's α ranged from 0.83 to 0.92 and composite reliability (CR) ranged from 0.84 to 0.93, indicating high internal consistency across all subgroups. The interpretation of related but distinct constructs was supported by the latent factor correlations within the PT subgroups, which ranged from 0.61 to 0.65. The items muscle tension, crying, agitation, and alertness were included in the first factor, while items related to movement, facial tension, and respiratory reaction were included in the second.

### 3.4 Survey Responses

To supplement the psychometric analyses, a short questionnaire was administered to 11 NICU professionals, including a neonatologist, two nurse practitioners, six NICU nurses, and two music therapists. The NICU professionals differed in their interpretations of the results. Some described the items as general “discomfort,” while others linked them to “pain” or “stress.” Several respondents associated more than one construct with the same factor. This lack of explicit agreement demonstrates that, in clinical practice, it is difficult to distinguish pain from stress based on behavioral cues alone. The detailed responses of NICU professionals are presented in Figure S1.

## 4 Discussion

Our study aimed to assess the dimensionality of the COMFORTneo scale by stratifying the sample based on gestational age and ventilation status. A consistent, unidimensional structure emerged in both the ventilated and non‐ventilated subgroups of the EPT, regardless of ventilation status. However, in the PT infants, both the ventilated and non‐ventilated subgroups showed a two‐factor structure. This demonstrated that the COMFORTneo scale does not measure a unidimensional construct in all subgroups.

The CFA confirmed these structural differences. The CFA model demonstrated mixed fit among EPT infants, with high CFI and TLI values and elevated RMSEA, suggesting little differentiation between behavioral indicators. It is more difficult to distinguish between pain and stress in this group due to neurological immaturity and a limited behavioral repertoire [1, 4]. As a result, distress forms often overlap in this group, consistent with the unidimensional structure identified in both EFA and CFA. PT infants, in contrast, showed consistently strong model fit indices, supporting a robust two‐factor structure [24]. Moderate correlations between these factors suggest that, although pain and stress behaviors are related, they are statistically distinct. This is consistent with research showing that more mature preterm infants exhibit increasingly differentiated distress signals [5, 27]. Similar multidimensional structures have been reported in other neonatal pain assessment tools, such as the Neonatal Pain, Agitation, and Sedation Scale (N‐PASS) and the original COMFORTneo scale [28]. For example, the N‐PASS distinguishes constructs related to pain, sedation, and agitation, while validation studies of the original COMFORTneo scale have identified separate factors for pain‐related behaviors and stress or discomfort‐related behaviors. These findings emphasize the need to interpret the COMFORTneo scale as a multidimensional tool in PT infants. The identified factor structure indicates that the scale captures more than one latent construct of distress, supporting the separation of pain and stress.

NICU professionals reported that it was often difficult to distinguish pain from stress in EPT infants. This observation aligns with previous studies highlighting the complexity of neonatal pain assessment [9, 29]. In contrast, experts noted clearer behavioral patterns in PT infants: muscle tension and facial expressions were more strongly associated with pain, while agitation and alertness were related to stress. These impressions are consistent with the two‐factor structure identified in the analyses, suggesting that COMFORTneo can serve as a multidimensional instrument, particularly in more mature newborns [22, 24]. The overlap between the statistical results and clinical impressions underlines the importance of developmentally sensitive interpretation. Clinicians may recognize subtle signs in EPT infants, but this often demands careful clinical judgment. Although the analyses suggest a unidimensional structure in this group, distinguishing between pain and stress at the bedside remains challenging, and the COMFORTneo scale alone is insufficient to achieve this distinction. Clinical decisions cannot rely solely on the behavioral scale. Contextual information is needed to interpret differences between subgroups of preterm infants [29]. It is important to recognize that in extremely preterm infants, stress and pain may be difficult to distinguish both behaviorally and in terms of subjective experience. While behavioral responses are observable and can be scored (e.g., via the COMFORTneo scale), they may reflect overlapping experiences of discomfort or distress. This suggests that even in PT infants, some behaviors may reflect overlapping constructs, highlighting the need for careful interpretation of factor assignments.

### 4.1 Strengths and Limitations

A strength of this study is the use of both factor analytic methods (EFA and CFA) together with input from NICU professionals. This combination allowed statistical validation to be placed in a practical context. Stratifying infants by gestational age and ventilation status also gave a more detailed view of structural differences within the preterm group. Limitations are the retrospective design, which may have left some influences unmeasured, and the small number of NICU professionals consulted, limiting the generalizability of the qualitative results. Even so, combining quantitative and qualitative approaches provides a valuable foundation for further work on the COMFORTneo scale.

### 4.2 Implications

These results have important implications for both EPT and PT infants. When applied carefully, the COMFORTneo scale can provide meaningful insights into infant well‐being. In PT infants, the scale can differentiate between two related but separate constructs, pain and stress, as supported by expert observations. In EPT infants, neurological immaturity and the limited range of behaviors make it difficult to separate pain from stress. In this group, total scores mainly reflect overall distress and should be interpreted with caution. Physiological measures (e.g., near‐infrared spectroscopy or electrodermal activity) might provide additional support [12]. At the same time, the choice of measures needs to take into account both construct specificity and practical feasibility. While statistical analyses indicate a unidimensional construct in extremely preterm infants, clinical experience may offer a different perspective, underscoring the importance of scoring approaches that are developmentally sensitive and informed by both NICU professionals and psychometric data.

## 5 Conclusion

In this study, we found a two‐factor structure in PT infants and a unidimensional structure in EPT infants using the COMFORTneo scale. These findings underline the need for developmentally sensitive interpretation of distress. Future research could explore additional methods to improve the assessment of pain and stress.

## Author Contributions

Mr. E. Koning conceptualized and designed the study, collected and analyzed the data, and drafted the first and final manuscript. Prof. EMW Kooi and Mr. AJ de Tempe conceptualized and designed the study, contributed to data interpretation, and critically reviewed and revised the manuscript. Prof A.F. Bos and Dr. NH van Dokkum conceptualized and designed the study, supervised data collection and analysis, contributed to interpretation of findings, and critically reviewed and revised the manuscript. All authors gave final approval of the version to be published and agree to be accountable for all aspects of the work.

## Funding

The authors have nothing to report.

## Ethics Statement

This study was conducted according to the guidelines for human studies in accordance with the World Medical Association Declaration of Helsinki. This study was approved by the Medical Ethics Committee of the University Medical Center Groningen.

## Conflicts of Interest

The authors declare no conflicts of interest.

## Supporting information


**Figure S1:** expert questionnaire participation of NICU healthcare professionals, presents the results of the expert questionnaire completed by NICU health professionals. The table summarizes how neonatologists, nurse practitioners, NICU nurses, and music therapists interpreted the COMFORTneo items in relation to the underlying constructs identified in the factor analyses (e.g., pain, stress, and discomfort). This material provides additional insight into the clinical interpretation of the identified factor structure.

## Data Availability

The data that support the findings of this study are available from the corresponding author upon reasonable request.

## References

[1] J. F. de Kieviet , L. Zoetebier , R. M. van Elburg , R. J. Vermeulen , and J. Oosterlaan , “Brain Development of Very Preterm and Very Low‐Birthweight Children in Childhood and Adolescence: A Meta‐Analysis,” Developmental Medicine and Child Neurology 54 (2012): 313–323. 22283622 10.1111/j.1469-8749.2011.04216.x

[2] C. Morgan , M. Davies‐Tuck , N. Marlow , et al., “Management and Outcomes of Extreme Preterm Birth,” BMJ (Clinical Research ed.) 376 (2022): e055924, 10.1136/bmj-2021-055924. PMC8744861 35012942

[3] M. Perry , Z. Tan , J. Chen , et al., “Neonatal Pain: Perceptions and Current Practice,” Critical Care Nursing Clinics of North America 30 (2018): 549–561, 10.1016/j.cnc.2018.07.013. 30447813 PMC6570422

[4] M. A. Ream and L. Lehwald , “Neurologic Consequences of Preterm Birth,” Current Neurology and Neuroscience Reports 18 (2018): 48, 10.1007/s11910-018-0862-2. 29907917

[5] B. O. Valeri , L. Holsti , and M. B. M. Linhares , “Neonatal Pain and Developmental Outcomes in Children Born Preterm: A Systematic Review,” Clinical Journal of Pain 31 (2015): 355–362. 24866853 10.1097/AJP.0000000000000114

[6] R. E. Grunau , L. Holsti , and J. W. B. Peters , “Long‐Term Consequences of Pain in Human Neonates,” Seminars in Fetal & Neonatal Medicine 11 (2006): 268–275. 16632415 10.1016/j.siny.2006.02.007

[7] K. M. Cook , J. De Asis‐Cruz , J. H. Kim , et al., “Experience of Early‐Life Pain in Premature Infants Is Associated With Atypical Cerebellar Development and Later Neurodevelopmental Deficits,” BMC Medicine 21 (2023): 435. 37957651 10.1186/s12916-023-03141-w PMC10644599

[8] N. H. Van Dokkum , M. L. A. de Kroon , P. H. Dijk , et al., “Course of Stress During the Neonatal Intensive Care Unit Stay in Preterm Infants,” Neonatology 119 (2022): 84–92. 34883490 10.1159/000520513

[9] A. Llerena , K. Tran , D. Choudhary , et al., “Neonatal Pain Assessment: Do We Have the Right Tools?,” Frontiers in Pediatrics 10 (2023): e1022751. 10.3389/fped.2022.1022751 PMC9932268 36819198

[10] D. Arabiat , E. Mörelius , K. Hoti , and J. Hughes , “Pain Assessment Tools for Use in Infants: A Meta‐Review,” BMC Pediatrics 23 (2023): 307. 37337167 10.1186/s12887-023-04099-7 PMC10278280

[11] S. Y. R. Fitri , L. Lusmilasari , M. Juffrie , and W. Rakhmawati , “Pain in Neonates: A Concept Analysis,” Anesthesia and Pain Medicine 9 (2019): e92455, 10.5812/aapm.92455. PMC6820293 31750094

[12] N. Witt , S. Coynor , C. Edwards , and H. Bradshaw , “A Guide to Pain Assessment and Management in the Neonate,” Seminars in Perinatology 40 (2016): 497–509. 27073748 10.1007/s40138-016-0089-y PMC4819510

[13] L. Glenzel , P. do Nascimento Oliveira , B. S. Marchi , et al., “Validity and Reliability of Pain and Behavioral Scales for Preterm Infants: A Systematic Review,” Pain Management Nursing 24 (2023): 84–96. 10.1016/j.pmn.2023.06.010 37524611

[14] K. Martakis , C. Hünseler , K. Thangavelu , A. Kribs , and B. Roth , “Pain‐Related Reactions Among Premature Infants With Gestational Age Less Than 26 Weeks: An Observational Cohort Study,” Neonatology 110 (2016): 261–266. 27299745 10.1159/000446172

[15] M. van Dijk , D. W. E. Roofthooft , K. J. S. Anand , et al., “Taking Up the Challenge of Measuring Prolonged Pain in (Premature) Neonates: The COMFORTneo Scale Seems Promising,” Clinical Journal of Pain 25 (2009): 607–616. 19692803 10.1097/AJP.0b013e3181a5b52a

[16] M. van Dijk , J. W. B. Peters , P. van Deventer , and D. Tibboel , “The COMFORTneo Scale: Development and Implementation in Neonates,” Neonatal Network 10 (2004): 7–13.

[17] K. S. Taber , “The Use of Cronbach's Alpha When Developing and Reporting Research Instruments in Science Education,” Research in Science Education 48, no. 6 (2018): 1273–1296, 10.1007/s11165-016-9602-2.

[18] M. Tavakol and R. Dennick , “Making Sense of Cronbach's Alpha,” International Journal of Medical Education 2 (2011): 53–55. 28029643 10.5116/ijme.4dfb.8dfd PMC4205511

[19] L. R. Fabrigar , D. T. Wegener , R. C. MacCallum , and E. J. Strahan , “Evaluating the Use of Exploratory Factor Analysis in Psychological Research,” Psychological Methods 4 (1999): 272–299.

[20] M. W. Watkins , “Exploratory Factor Analysis: A Guide to Best Practice,” Journal of Black Psychology 44 (2018): 1–21.

[21] J. B. Schreiber , “Issues and Recommendations for Exploratory Factor Analysis and Principal Component Analysis,” Research in Social & Administrative Pharmacy 17 (2021): 1004–1011. 33162380 10.1016/j.sapharm.2020.07.027

[22] T. A. Brown , Confirmatory Factor Analysis for Applied Research, 2nd ed. (Guilford Press, 2015).

[23] G. Zhang and K. J. Preacher , “Factor Rotation and Standard Errors in Exploratory Factor Analysis,” Journal of Educational and Behavioral Statistics 40 (2015): 579–603.

[24] L. Hu and P. M. Bentler , “Cutoff Criteria for Fit Indexes in Covariance Structure Analysis: Conventional Criteria Versus New Alternatives,” Structural Equation Modeling 6 (1999): 51–55.

[25] S. Kolenikov and K. A. Bollen , “Testing Negative Error Variances: Is a Heywood Case a Symptom of Misspecification?,” Sociological Methods & Research 41 (2008): 124–167, 10.1177/0049124112442138.

[26] B. Thompson , Exploratory and Confirmatory Factor Analysis: Understanding Concepts and Applications (American Psychological Association, 2004).

[27] E. Cignacco , J. P. H. Hamers , L. Stoffel , et al., “Pain Assessment in Premature Infants: A Clinical Perspective,” Neonatal Intensive Care 14 (2001): 31–36.

[28] M. E. Morgan , S. Kukora , M. Nemshalk , and C. J. Shuman , “Neonatal Pain, Agitation, and Sedeation Scale's Use, Reliability and Validity: A Systematic Review,” Journal of Perinatology 40 (2020): 1753–1763, 10.1038/s41372-020-00840-7. 33009491

[29] H. M. Obeidat , D. A. Dwairej , and A. S. Aloweidi , “Pain in Preterm Infants: Different Perspectives,” Journal of Perinatal Education 30 (2021): 185–195. 34908817 10.1891/J-PE-D-20-00032 PMC8663768

