# Supplementary material for: A Factor Analysis of the COMFORTneo Scale: What Are We Measuring?
Source: Paediatr Neonatal Pain. 2026 May 14;8(2):e70033. doi: 10.1002/pne2.70033 (PMC13173448; doi:10.1002/pne2.70033)
Supplement: Supplementary file 1 — Figure S1: expert questionnaire participation of NICU healthcare professionals, presents the results of the expert questionnaire completed by NICU health professionals. The table summarizes how neonatologists, nurse practitioners, NICU nurses, and music therapists interpreted the COMFORTneo items in relation to the underlying constructs identified in the factor analyses (e.g., pain, stress, and discomfort). This material provides additional insight into the clinical interpretation of the identified factor structure. [file PNE2-8-e70033-s001.docx]

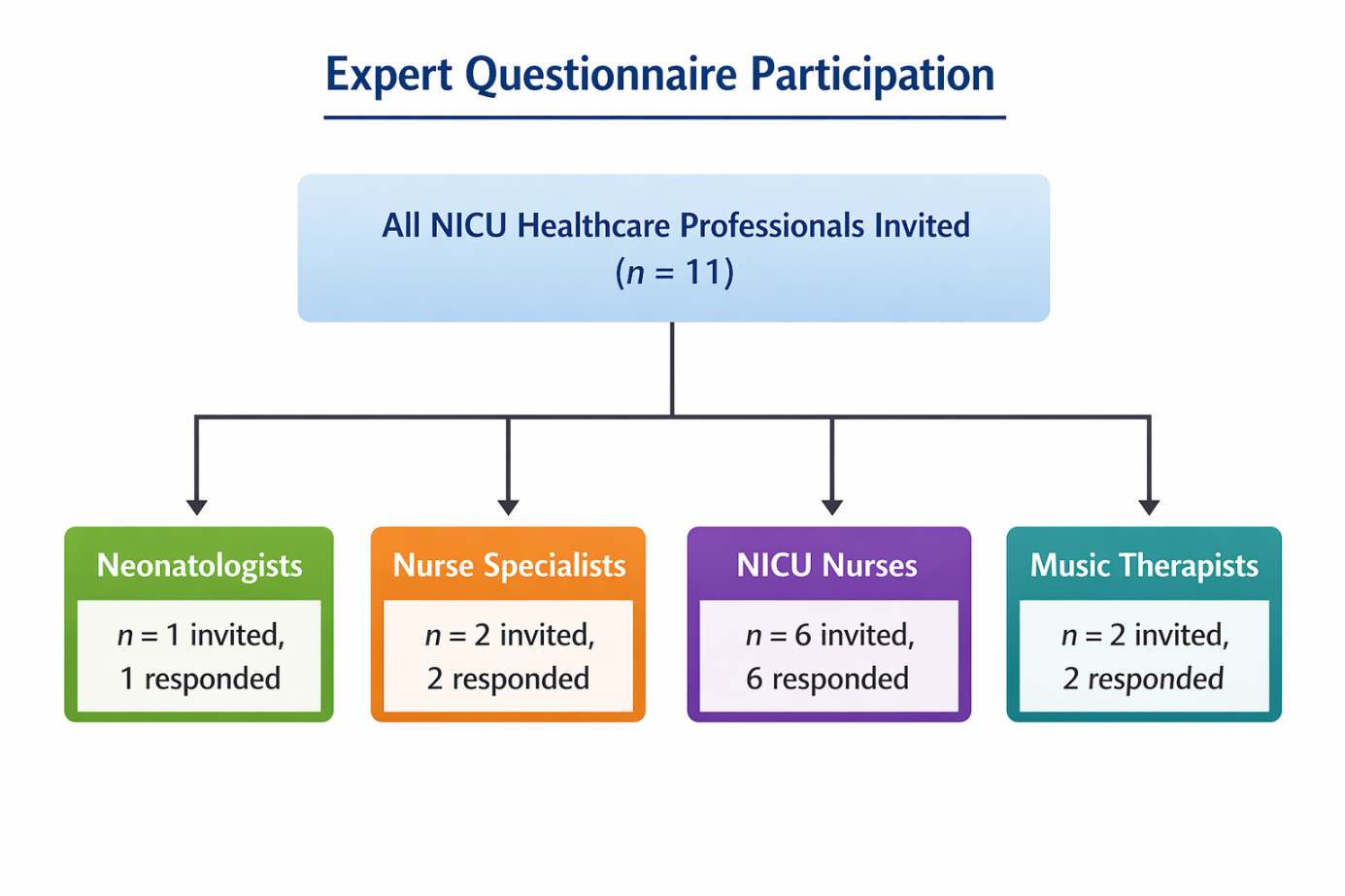


**Supplementary Figure 1. expert questionnaire participation of NICU healthcare professionals**
